# Intermolecular RNA Recombination Occurs at Different Frequencies in Alternate Forms of Brome Mosaic Virus RNA Replication Compartments

**DOI:** 10.3390/v10030131

**Published:** 2018-03-15

**Authors:** Hernan Garcia-Ruiz, Arturo Diaz, Paul Ahlquist

**Affiliations:** 1 Institute for Molecular Virology, University of Wisconsin-Madison, Madison, WI 53706, USA; hgarciaruiz2@unl.edu (H.G.-R.); adiaz@lasierra.edu (A.D.); 2 Nebraska Center for Virology, Department of Plant Pathology, University of Nebraska-Lincoln, Lincoln, NE 68503, USA; 3 Department of Biology, La Sierra University, Riverside, CA 92515, USA; 4 Howard Hughes Medical Institute and Morgridge Institute for Research, University of Wisconsin-Madison, MadisonWI 53706, USA

**Keywords:** Brome mosaic virus (BMV), RNA recombination, viral RNA replication compartments

## Abstract

Positive-strand RNA viruses replicate their genomes in membrane-bound replication compartments. Brome mosaic virus (BMV) replicates in vesicular invaginations of the endoplasmic reticulum membrane. BMV has served as a productive model system to study processes like virus-host interactions, RNA replication and recombination. Here we present multiple lines of evidence showing that the structure of the viral RNA replication compartments plays a fundamental role and that recruitment of parental RNAs to a common replication compartment is a limiting step in intermolecular RNA recombination. We show that a previously defined requirement for an RNA recruitment element on both parental RNAs is not to function as a preferred crossover site, but in order for individual RNAs to be recruited into the replication compartments. Moreover, modulating the form of the replication compartments from spherular vesicles (spherules) to more expansive membrane layers increased intermolecular RNA recombination frequency by 200- to 1000-fold. We propose that intermolecular RNA recombination requires parental RNAs to be recruited into replication compartments as monomers, and that recruitment of multiple RNAs into a contiguous space is much more common for layers than for spherules. These results could explain differences in recombination frequencies between viruses that replicate in association with smaller spherules versus larger double-membrane vesicles and convoluted membranes.

## 1. Introduction

Viruses are intracellular parasites that depend on host cells for replication [1,2,3]. Viral infection induces cellular responses such as adaptive immunity and RNA silencing [4,5] that impose constant selection pressure on the virus. RNA recombination is the formation of novel sequences by joining noncontiguous RNA segments from one (intramolecular) or more (intermolecular) molecules resulting in rapid modification of viral genomes and contributing to RNA virus evolution, short-term variability and survival [6,7,8]. RNA recombination has been proposed to occur by several mechanisms, including primer extension, breakage and re-ligation, and template switching [8]. Although primer extension has been implicated [9], Brome mosaic virus (BMV) RNA recombines mainly by template switching [6,7,10,11] in which the polymerase initiates RNA synthesis on one template (donor), stops, changes template and continues synthesis on a second template (acceptor). RNA sequences that participate in the formation of crossover sites at high frequency are referred to as preferred recombination sites [8]. The essential components of template switching are two parental RNAs (donor and acceptor) and the viral RNA polymerase [7,8,10,11]. Consistent with this model, both viral and host components affecting abundance and features of viral RNA influence both the frequency of RNA recombination and features of the progeny [7,9,12,13,14,15,16]. Cellular components with the biggest impact are proteins involved in host RNA metabolism/degradation, RNA binding, or RNA silencing [15,16,17,18]. Similarly, viral RNA sequences and mutations affecting polymerase properties influence the distribution of crossover sites [11,19]. Less is known about the role of the structure of the viral replication compartments in viral RNA recombination.

Positive-strand RNA viruses of plants and animals replicate their genomic RNA on remodeled intracellular membranes. For many such viruses, this genome replication occurs in virus-induced, vesicular RNA replication compartments (spherules) bound to intracellular membranes of the endoplasmic reticulum, peroxisomes, mitochondria or chloroplasts [20,21,22,23,24]. Spherules function as mini-organelles whose purpose is to produce more copies of the viral genome [20,21,22].

**Figure 1:**
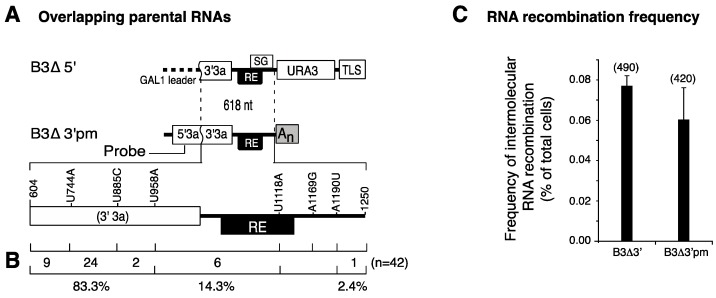
Distribution of crossover sites after one yeast generation in brome mosaic virus (BMV) RNA3. Single lines represent non-coding regions, and labeled boxes represent 3a, URA3, the template recruitment element (RE), the subgenomic RNA4 promoter (SG), and the 3′ tRNA-like sequence (TLS). (**A**) Recombination partners B3Δ5′ and B3Δ3′pm are non-replicatable, overlapping (~618 nt) B3URA3 derivatives transcribed in vivo from plasmids carrying the *CUP1* or *GAL1* promoter, respectively. In B3Δ5′ the 5′UTR and the 5′ half of 3a was replaced by the *GAL1* leader sequence. B3Δ3′pm harbored point mutations and its 3′end is formed by the *ADH1* polyadenylation signal (An). A probe against 5′3a sequences was used to detect intermolecular recombinants; (**B**) The number and frequency (%) of recombinants formed between point mutations. Forty-two RNA recombinants randomly selected from three biological replicates were sequenced; (**C**) Frequency of intermolecular RNA recombination between B3Δ5′ and B3Δ3′ or B3Δ3′pm. Bars represent the average and standard error of three replicates. In parenthesis is the total number of cases observed. The average sample size per treatment was 210,000 cells.

**Figure 2:**
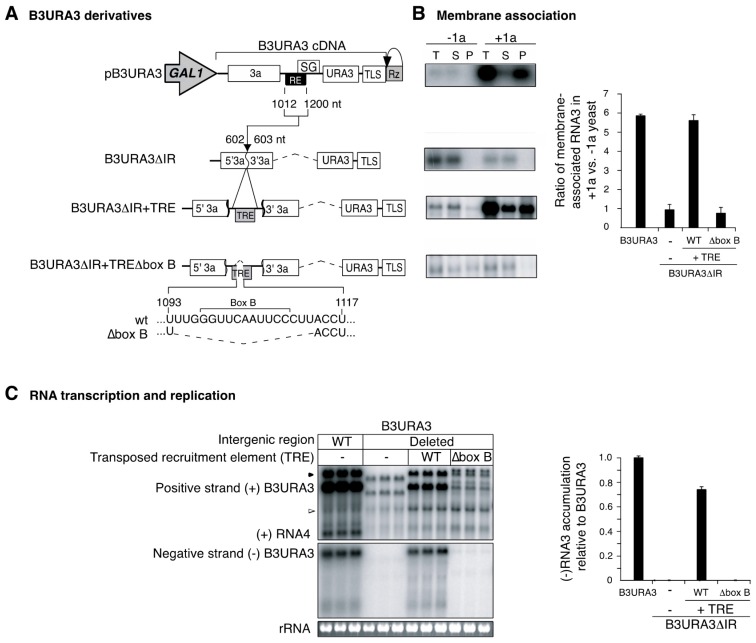
A transposed template recruitment element (RE) supports BMV RNA3 recruitment and replication. (**A**) Cassettes for in vivo transcription of B3URA3 and derivatives. The bracketed region represents a cDNA copy of B3URA3 flanked by a 5′-linked *GAL1* promoter and a 3′-linked, self-cleaving, hepatitis delta ribozyme (Rz). Coordinates correspond to wt BMV RNA3. In B3URA3ΔIR, a B3URA3 derivative lacking the intergenic region, RE sequences (nt 1012–1200) were inserted between nt 602 and 603, creating B3URA3ΔIR + TRE. A box B-deficient TRE (B3URA3ΔIR + TREΔbox B) was created by removing nt 1094–1113; (**B**) Membrane association of B3URA3 derivatives in yeast expressing or lacking BMV replication protein 1a. After transcription for 72 h, equal amounts of cells were spheroplasted and lysed osmotically. Half of the lysate was processed to obtain the total RNA fraction (T). The other half was centrifuged at 10,000× *g* to yield a pellet (P) and supernatant (S) fractions. RNA was isolated from each fraction, and equal proportions analyzed by Northern blotting to detect positive-strand URA3 sequences. Representative blots are shown. The histogram shows the average and standard error from three replicates; (**C**) RNA replication in the absence of selection. Yeast was transformed with plasmids carrying B3URA3, or its derivatives, and with plasmids expressing 1a and 2a^pol^ replication proteins. Transcription was induced with galactose for 72 h, and equal amounts of cells harvested for RNA extraction. Equal amounts of total RNA were analyzed by Northern blotting with a single-stranded, 32-P labeled RNA probe complementary to positive (+)- or negative (−)-strand URA3. The solid arrowhead points to transcripts that have not been cleaved by the ribozyme. The empty arrowhead points to short transcripts formed after premature termination of transcription at the oligo(A) tract (Sullivan and Ahlquist, 1999). Ethidium bromide staining of 18S rRNA is indicated at the bottom. Negative-strand B3URA3 accumulation was quantified for three biological replicates. The histogram shows average and standard error relative to B3URA3.

BMV is a member of the alphavirus-like superfamily of human, animal, and plant positive-strand RNA viruses. BMV encodes three genomic RNAs (RNA1, RNA2 and RNA3) and one sub-genomic mRNA, RNA4. RNA1 and RNA2 encode the non-structural replication proteins 1a and 2a^pol^, respectively. 1a is a multifunctional protein that interacts with host proteins to induce the invagination of endoplasmic reticulum membranes to form spherules, recruits genomic viral RNA and the 2a^pol^ to replication compartments and provides helicase and RNA capping functions [20]. 2a^pol^ is the RNA-dependent RNA polymerase. RNA3 encodes 3a and the single capsid protein, both of which are required for systemic movement in plants [25]. While 3a is translated from RNA3, the capsid protein is expressed from subgenomic RNA4. The RNA3 intergenic region is ~250 nt long and harbors both the promoter for transcribing RNA4 from a negative-strand template (Figure 1A) and the *cis*-acting template recruitment element, which is ~188 nt long (Figure 2) [26] and directs the 1a-mediated recruitment of positive-strand RNA3 to BMV RNA replication compartments [26,27].

Although first isolated from brome grass, BMV replication, encapsidation, and recombination have been reconstituted in the yeast *Saccharomyces cerevisiae* by expressing viral replication and encapsidation proteins in combination with one or more genomic RNAs [10,28,29,30]. This system recapitulates all fundamental aspects of BMV replication in plants, including parallel dependence on the same viral proteins, viral protein-membrane interactions, *cis*-acting RNA replication and transcription signals, and host factors [20,27,31,32,33,34].

In plants, BMV recombination occurs by template switching both during positive- and negative-strand RNA synthesis [7]. We developed a yeast system to induce BMV intermolecular RNA3 recombination by template switching during negative strand RNA synthesis, which allows reproducible measurements of the frequency of intermolecular RNA recombination per cell [10]. For BMV and other positive-strand RNA viruses, RNA replication occurs in virus-induced, membrane-bounded compartments [20,21,35,36]. Spherular invaginations are prominent features of BMV RNA replication in natural plant infections and yeast, as well as in many other plant, insect and animal positive-strand RNA viruses [21,22,36]. Interestingly, modulating the expression of BMV replication proteins 1a and 2a^pol^ in yeast leads to the formation of large, karmellae-like, multi-level stacks of appressed double membranes referred to as layers [37]. Spherules and layers support BMV replication to similar levels [37]. Although there are no published reports of layer-like structures in plants, the BMV-induced layers resemble the convoluted membranes induced by noroviruses and picornaviruses [38,39]. 

Since RNA recombination is linked to replication and requires two or more RNA templates to be within reach of the viral RNA polymerase [6,7], we hypothesized that the frequency of intermolecular RNA recombination might differ between the small spherules and more expansive membrane layers. To test this hypothesis, our experiments were based on two features of BMV RNA replication: recruitment of BMV genomic RNA into replication compartments is mediated by 1a interacting with *cis*-acting sequences (the recruitment element) in the viral RNA, and alternate forms of the replication compartments (spherules or layers) can be induced by modulating expression of replication proteins 1a and 2a^pol^ [27,37]. Mutational inactivation of the recruitment element abolished intermolecular RNA recombination. However, adding a recruitment element at a transposed location restored RNA combination. Mapping of crossover sites using point mutations and structurally different parental RNAs showed that the recruitment element is not required as a preferential crossover site, but rather for its role in recruiting monomeric parental RNAs into the replication compartments. Furthermore, modulating the shape of the RNA replication compartments from spherules to layers dramatically increased intermolecular RNA recombination rates by 200- to 1000-fold. Thus, expanding the physical size of the RNA replication compartments beyond their normal size relieved the limiting constraint on intermolecular RNA recombination. 

Our results support a model in which BMV genomic RNAs are individually recruited into replication compartments, and intermolecular recombinants are formed in replication compartments receiving multiple genomic RNAs. Compartmentalization of RNA replication in spherules and recruitment of parental RNAs to a common spherular replication compartment impose a limiting constraint to intermolecular RNA recombination. That constraint is removed by the contiguous space of layers. 

## 2. Materials and Methods 

### 2.1. Yeast Methods

Yeast strain YPH500 *(MATα ura3-52 lys2-801 ade2-101 trp1-63 his3-200 leu2-1)* was used in all experiments. Cultures were grown at 30 °C in defined synthetic medium containing 2% galactose or 2% glucose. Relevant amino acids were omitted to maintain selection for DNA plasmids. When necessary, uracil was omitted to select for BMV-replication dependent *URA3* expression. Transformation with DNA plasmids was done using the lithium acetate-polyethylene glycol method [40].

### 2.2. Plasmids

BMV replication proteins 1a and 2a^pol^ were expressed from the *ADH1* promoter using pB1CT19 or pB2CT15, expressing the *HIS3* and *LEU2* selectable markers, respectively [28]. Where indicated, 1a and 2a^pol^ were expressed from the *GAL1* promoter using pB1YT3H or pB2YT5 as described [10]. When either was omitted, pRS313 (*HIS3*) or pRS315 (*LEU2*) “empty” markers [41] were transformed into yeast to grow all cultures in the same selective medium. Plasmids that express BMV RNA3 derivatives were based on pB3URA3, which contains a full length RNA3 cDNA between the *GAL1* promoter and a self-cleaving hepatitis delta virus ribozyme followed by the *ADH1* polyadenylation signal, and with the coat gene replaced by *URA3* (Figure 1A) [29]. pB3URA3 is a yeast *CEN4* centromeric plasmid containing the *TRP1* marker gene. Plasmid pB3**∆**5′ (pHGR1) is a pB3URA3 derivative used to transcribe B3**∆**5′ (Figure 1B) [10]. Plasmids expressing B3**∆**3′ (pHGR3)(Figure 1B) [10] and its derivatives from the *CUP1* promoter had the *LYS2* marker instead of *TRP1* to simultaneously select for two RNA3-encoding plasmids. No empty *TRP1* or *LYS2* markers were used when RNA3-encoding plasmids were omitted.

### 2.3. Plasmid Construction

Standard procedures were used for all DNA manipulations [42]. When necessary, restriction fragments with 5′ overhanging ends were filled in using the Klenow fragment of DNA polymerase I and 3′ overhanging restriction fragment ends were blunt ended by treating with T4 DNA polymerase. The overall structure and sequence of the plasmids was confirmed by restriction digest and sequencing. Laboratory designations for plasmids are given in parenthesis.

**pB3URA3-Vector (pHGR82).** pHGR82 is a pUC119-based version of pB3URA3 for cloning purposes. To make pHGR82, the EcoRI-PstI fragment of pHGR11 was replaced with the equivalent fragment from pB3URA3 [29].

**pB3URA3pm (pHGR83.9).** Random PCR mutagenesis [42] was used to introduce point mutations into pB3URA3 creating pB3URA3pm. A PCR insert was generated using pB3URA3 as template and oligonucleotides 325 [d(GTATGATAAAGGAGAG)] and 1976 [d(GGTTCCTTTGTTACTTCTTCTGCCGCCTGCTTCAAACCGCT)]. After ClaI-BglII digestion, the PCR product was used to replace the corresponding fragment on pHGR82, creating pHGR82 derivatives with point mutations (pHGR82pm) along the 3a ORF and the intergenic region. To make pHGR83.9, the EcoRI-PstI fragment of pHGR82pm was used to replace the equivalent fragment on pB3URA3.

**pCUP1-B3URA3 (pHGR41).** Is a pB3URA3 derivative to express B3URA3 from the *CUP1* promoter. The EcoRI-ClaI fragment from pHGR3 replaced the equivalent fragment on pB3URA3 [29].

**pB3∆3′pm p(HGR100.9).** The ClaI-AfeI fragment of pB3∆3′ [10] was replaced with the equivalent fragment from pHGR83.9.

**pB3URA3∆IR (pB3HGR27)**. The EcoRI-PstI fragment of pB3URA3 [29] was replaced with the equivalent fragment from pHGR11 [10], generating a B3URA3 derivative that lacks the entire intergenic region.

**pB3∆3′ + TRE (pB3HGR53).** BMV genomic RNA3 intergenic region sequences (nt 1012–1200) needed for full 1a-mediated recruitment of RNA3 templates to replication compartments [26] were inserted at the ClaI site of pB3∆3′ [10]. The insert was generated by PCR using pB3URA3 [29] as template and oligonucleotides 3261 [d(CCATCGATAGACGCGTGG-TCTAACAAGCTCGGTC)] and 3014 [d(CCATCGATACGCGTAATAATAACTCAGACA-CAC)]. Both oligonucleotides introduce a ClaI restriction site for cloning purposes. Additionally, oligonucleotide 3261 introduces a stop codon to resemble the natural 3a stop codon. The PCR fragment was ClaI-digested before ligating it to a ClaI-digested pB3∆3′.

**pB3∆3′∆box B + TRE (pB3HGR54).** The TRE was inserted as described for pB3HGR53 using pB3∆3′∆box B (pHGR22) [10] as the vector.

**pB3∆3′∆IR + TRE (pB3HGR55).** The TRE was inserted as described for pB3HGR53 using pB3∆3′∆IR (pHGR7) [10] as the vector.

**pB3URA3 + TRE (pB3HGR62).** The RE was inserted as described for pB3HGR53 using pB3URA3 [29] as the vector.

**pB3URA3∆3′3a (pB3HGR63).** The AfeI-AfeI fragment from pB3HGR62 was removed and the vector religated. The resulting intergenic region is a hybrid consisting of pieces from the wt intergenic region and from the transposed RE. Plasmid B3HGR63 lacks nt 603–1003 (3′3a) of the wt BMV genomic RNA3.

**pB3URA3 + TRE∆box B (pB3HGR65).** A box B-deficient transposed RE (TRE∆box B) was generated by PCR using pB3HGR22 [10] as template and olignucleotides 3261 and 3014. The PCR fragment was ClaI-digested before ligating it to a ClaI-digested pB3URA3 [29]. 

**pB3URA3∆IR + TRE (pB3HGR68).** The ClaI-ClaI fragment of pHGR62, containing the transposed RE, was inserted at the ClaI site of pHGR27.

**pB3URA3∆IR + TRE∆box B (pB3HGR69).** The ClaI-ClaI fragment of pHGR65, containing a box B-deficient transposed RE, was inserted at the ClaI site of pHGR27.

**pB3∆3′ + TRE∆box B (pB3HGR85).** The ClaI-ClaI fragment of pHGR65, containing a box B-deficient transposed RE, was inserted at the ClaI site of pHGR3 [10].

**pB3∆3′∆box B + TRE∆box B (pB3HGR86).** The ClaI-ClaI fragment of pHGR65, containing a box B-deficient transposed RE, was inserted at the ClaI site of pB3∆3′∆Box B (pHGR22) [10].

**pB3∆3′∆IR + TRE∆box B (pB3HGR87).** The ClaI-ClaI fragment of pHGR65 was inserted at the ClaI site of pB3∆3′∆IR (pHGR7) [10].

**pCUP1-B3∆5′ (pHGR140).** It is a pB3URA3 derivative that lacks nt 1 to 602. There is no *GAL1* leader, the *CUP1* promoter was directly fused to RNA3 sequences. The SnaBI-ClaI fragment of pHGR41 was removed and the vector religated after blunting the ClaI end.

### 2.4. Induction of Transcription and Screening for Ura^+^ Cells

Transient induction of transcription from the *GAL1* or the *CUP1* promoters and screening for cells that acquired the ability to grow (plasmid launching) in the absence of uracil (Ura^+^ cells) was performed as described [10]. Induction cultures (8 mL) were inoculated to an A_600_ optical density (OD) of 0.002. In vivo transcription of parental RNAs was modulated by controlling the source of carbon (2% glucose or 2% galactose) and the amount of copper (zero or 500 μM CuSO_4_) in uracil-amended liquid media. Transcription of B3∆5′ from pB3∆5′ [10] was induced by galactose, while transcription of B3∆3′ from pB3∆3′ was induced with copper. After 72 h (~9 to 10 generations), transcription was repressed by diluting all cultures to an OD A_600_ of 0.1 in one mL of media lacking uracil and copper, and with glucose as the only carbon source. Repression cultures were incubated for 2 h and further diluted two (OD = 0.05) and 125- fold (OD = 0.00095) for plating purposes. Ura^+^ cells were identified by selection on solid media lacking uracil. For every induction culture, 1 mL of the two-fold diluted repression culture was spread onto 10 plates lacking uracil, at a rate of 100 µL per plate. In parallel, 200 µL of the final dilution was spread onto two uracil-amended plates to determine the total number of cells in the sample. Selection for the plasmids encoding BMV replication proteins 1a and 2a^pol^ was maintained at all times and the number of colonies counted after 72 h. These conditions resulted on an average sample size of 210,000 cells. For experiments in which cells were grown under layer-forming conditions, the sample size was reduced to 700 cells, and the selection plates contained galactose but lacked histidine and lysine to maintain selection for expression of 1a and 2a^pol^.

### 2.5. Identification of Intermolecular RNA Recombinants by Northern Blotting

Induction cultures and Ura^+^ colonies obtained after one yeast generation were individually grown in liquid cultures (8 mL) containing or lacking uracil, respectively. Total RNA extraction and northern blot analysis were performed as described in [28]. Ura^+^ colonies obtained in all other recombination experiments were individually grown in one mL of media lacking uracil in 96-well plates. Total RNA extraction was performed as described elsewhere [43]. DNA-dependent RNA transcripts, replication products, and RNA recombinants were detected with strand-specific ^32^P-labeled RNA probes transcribed in vitro and targeting URA3 or 3a sequences (5′3a or 3′3a) (Figure 1). The frequency of homologous intermolecular RNA recombination was measured as the ratio of the number of Ura^+^ cells that harbored an intermolecular RNA recombinant to the total number of cells present in the sample.

### 2.6. Expected Intermolecular RNA Recombination Frequency 

To map the distribution of crossover sites, acceptor RNAs harboring point mutations or structurally different were used in combination with a common donor RNA. The expected frequency of intermolecular RNA recombinants was estimated for four possible formation of crossover sites determined by the homology between parental RNAs: (1) exclusively within 3′3a sequences; (2) exclusively from the natural to the transposed recruitment element; (3) exclusively within the natural recruitment elements; and (4) randomly. Random formation of crossover sites was weighted for the length of 3′3a sequences (410 nt) and the recruitment element (188 nt).

### 2.7. RNA Recombination after One Yeast Generation

The experiment was conducted as described above, except that transcription was transiently induced for only 15 h and cultures were started at an OD of 0.3.

### 2.8. Cell Fractionation

Yeast cells were grown in synthetic galactose medium to mid-log phase for 72 h. Spheroplasting, cell fractionation and RNA extraction were performed as described elsewhere [27].

### 2.9. RT-PCR Cloning and Sequencing of Intermolecular RNA Recombinants

Three micrograms of DNAse I-treated total RNA were used as substrate for reverse transcription, PCR amplification, cloning and sequencing as described previously [10].

### 2.10. Electron Microscopy

After transforming with the appropriate plasmids, cells were grown under induction conditions and prepared for electron microscopy as described [37].

### 2.11. Western Blotting

Total protein extraction and immuno-detection of 1a and 2a^pol^ in yeast liquid cultures was performed as described [44].

## 3. Results

### 3.1. The RNA3 Recruitment Element Is Not a Preferred Recombination Site 

Prior work showed that intermolecular RNA recombination of BMV RNA3 requires the recruitment element, including a bulged stem loop known as the box B, in both parental RNAs [10].

Two nonexclusive hypotheses can explain this requirement: the recruitment element is needed solely for individual recruitment of each parental RNA into replication compartments; and/or formation of crossover sites preferentially occurs within the recruitment element. To distinguish between these possibilities, we introduced single nucleotide substitutions in one of the parental RNAs (B3Δ3′pm) and mapped the distribution of crossover sites in the progeny (Figure 1). Parental RNAs B3Δ5′ and B3Δ3′pm were co-expressed in the presence of BMV replication proteins 1a and 2a^pol^. B3Δ5′ and B3Δ3′pm are partially overlapping and unable to replicate (Figure 1B). Thus, only cells harboring an RNA3 replicon formed through recombination were expected to grow and form Ura^+^ colonies [10]. To reduce the likelihood of processing siblings, Ura^+^ cells were selected after one yeast generation and individually grown before extracting total RNA. A probe (5′3a) specifically targeting negative-strand B3∆3′ was used to distinguish intermolecular B3URA3 RNA recombinants from intramolecular sURA3 RNAs resulting from B3∆5′ (Figure 1B) [10].

The marked acceptor RNA, B3Δ3′pm, participated in intermolecular RNA recombination at a frequency similar to that of wild type B3Δ3′ (Figure 1D). Forty-two recombinants from three biological repetitions were sequenced and the distribution of the point mutations was mapped (Figure 1C). Most of the crossover sites (~83%) occurred within 3a sequences common to B3Δ5′ and B3Δ3′pm. The area with the highest frequency (62%) of crossover sites was between nt 744 and 958 on the 3a ORF. In contrast, only 14.3% of the progeny resulted from crossover events between markers 959 and 1118, which include most of the recruitment element and part of 3a (Figure 1C). These results show that the recruitment element is not a preferred crossover site. 

### 3.2. RNA3 Recruitment into Replication Compartments Through a Transposed Recruitment Element

**Figure 3:**
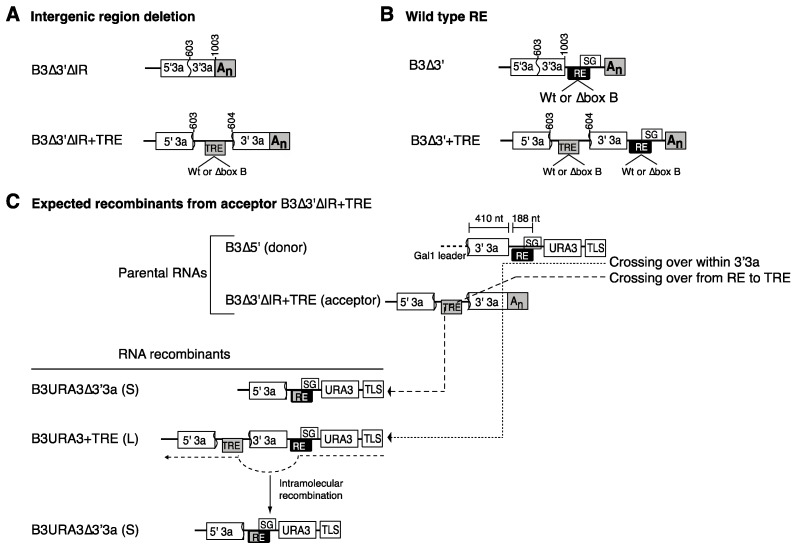
Diagram of parental RNAs and expected recombinants. Non-replicatable B3∆3′ derivatives were transcribed in vivo from the *CUP1* promoter as recombination partners of B3∆5′. Labeled boxes are as in Figure 1A. (**A**) In B3∆3′ the recruitment element (RE) was removed by deleting the intergenic region (∆IR), inactivated by a box B deletion (∆box B), or (**B**) is wild type. A wild type or a box B-deficient transposed recruitment element (TRE) was provided to all B3∆3′ derivatives. (**C**) Expected progeny from recombination between B3∆5′ and an acceptor lacking the intergenic region and harboring a TRE. Crossover sites (indicated by a dashed line with an arrow head) within the common 3′3a area (~410 nt) would form B3URA3 + TRE (L), and subsequent intramolecular recombination from RE to TRE would form B3URA3Δ3′3a. Intermolecular RNA recombination within RE (~188 nt long) and TRE sequences can form B3URA3Δ3′3a.

Results described above (Figure 1C) and before [10] suggest that in the process of RNA recombination the role of the recruitment element is to mediate 1a-dependent recruitment of individual genomic RNAs into the replication compartments. This model predicts that RNAs, such as parental B3∆3′ derivatives lacking the intergenic region, would participate in intermolecular RNA recombination if recruited into RNA replication compartments by an alternate route. We developed a strategy for recruiting RNA3 derivatives to RNA replication compartments independently of the natural recruitment element. The RNA3 recruitment element was inserted at a transposed location in the middle of the 3a ORF in B3URA3 (Figure 2A) and in parental B3∆3′ derivatives (Figure 3A,B). The transposed recruitment element (TRE) consists of BMV RNA3 nt 1012–1200 [26] and lacks the subgenomic promoter [26]. Template recruitment (Figure 2B) and RNA replication (Figure 2C) were reduced to background levels after removing the intergenic region from B3URA3 (B3URA3∆IR). However, addition of the transposed recruitment element (B3URA3∆IR + TRE), but not of a box B-deficient transposed recruitment element (B3URA3∆IR + TRE∆box B), restored template recruitment (Figure 2B) and RNA replication (Figure 2C). Accordingly, the recruitment element is functional in the middle of the 3a ORF and was added to parental RNA B3∆3′ derivatives (Figure 3A,B) to provide an alternate mode of 1a-mediated recruitment into the RNA replication compartments.

### 3.3. Intramolecular RNA Recombination Occurs at High Frequency 

**Figure 4:**
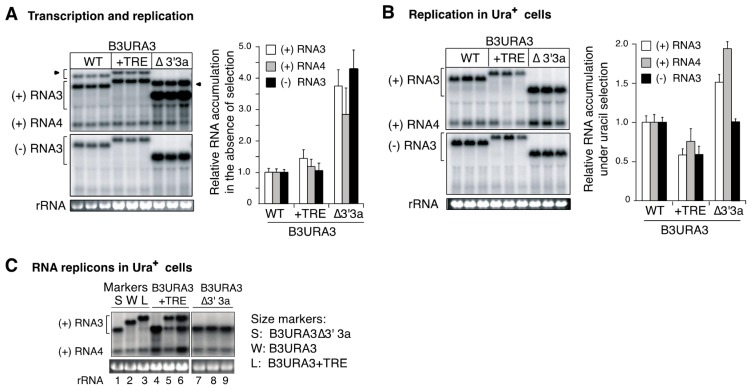
Replication of B3URA3 + TRE and B3URA3Δ3′3a, and intramolecular RNA recombination in spherule-forming conditions. (**A**) Accumulation of B3URA3 + TRE and B3URA3Δ3′3a in actively transcribing cultures in the absence of selection. In yeast expressing BMV 1a and 2a^pol^, B3URA3 or its derivatives were transcribed from the GAL1 promoter in liquid cultures for 72 h (9–10 yeast generations). Equal amounts of cells were harvested for RNA extraction and analysis by Northern blotting with 32-P labeled, strand-specific URA3 probes. Representative Northern blots are shown. Arrowheads indicate transcripts before ribozyme cleavage. RNA3 and RNA4 of positive and negative polarity are indicated are indicated by (+) and (−), respectively. Ethidium bromide staining of 18S rRNA is indicated at the bottom. The histogram shows RNA accumulation relative to B3URA3, and bars represent the average and standard error of six biological replicates. B3URA3Δ3′3a accumulated to higher levels than B3URA3 and B3URA3 + TRE (*p* < 0.01); (**B**) Accumulation of B3URA3 + TRE and B3URA3Δ3′3a in Ura^+^ cells. Individual Ura^+^ colonies were grown in liquid cultures (8 ml) under uracil selection. Total RNA was extracted and processed as in (A). Positive strand RNA3 and RNA4 in B3URA3Δ3′3a accumulated to higher levels than B3URA3 and B3URA3 + TRE (*p* < 0.01). (**C**) Detection of intramolecular RNA recombinants in Ura^+^ colonies after plasmid launching of B3URA3 + TRE. B3URA3 and B3URA3Δ3′3a were processed in parallel as size markers. For each construct, twenty Ura^+^ colonies obtained after plasmid launching were individually grown in liquid cultures (8 mL) lacking uracil, the RNA extracted, and analyzed as in (A). Lane 4 shows B3URA3Δ3′3a replacing B3URA3 + TRE. Lanes 5 and 6 show both B3URA3 + TRE and B3URA3Δ3′3a. B3URA3Δ3′3a did not suffer genetic modifications (lanes 7, 8 and 9). Ethidium bromide staining of 18S rRNA is indicated at the bottom.

Recombination between donor B3∆5′ and acceptor B3∆3′ derivatives lacking the intergenic region and harboring a transposed recruitment element is predicted to form two groups of structurally different replicons (Figure 3C and Figure 4A). Crossover events within the common 3′3a regions of B3∆5′ and B3∆3′ derivatives lead to the formation of a long recombinant replicon, termed B3URA3 + TRE (Figure 3C). However, crossover events between the recruitment element of B3∆5′ and the transposed recruitment element of B3∆3′ derivatives lead to the formation of short recombinant replicon, termed B3URA3∆3′3a (Figure 3C,D). Additionally, B3URA3∆3′3a could be formed from B3URA3 + TRE by intramolecular RNA recombination within natural and transposed recruitment element sequences (Figure 3C). Thus, we measured the frequency of intramolecular RNA recombination in B3URA3 + TRE and determined its genetic stability. We also compared the replication of B3URA3 + TRE to B3URA3∆3′3a. Replication of B3URA3 + TRE and B3URA3∆3′3a was initiated by plasmid launching and Ura^+^ colonies were selected [29]. B3URA3 was included in parallel for comparison.

In the absence of selection and continued RNA3 cDNA plasmid transcription, B3URA3 + TRE accumulated to similar levels as B3URA3 (Figure 4A). After repressing plasmid transcription and under uracil selection, B3URA3 + TRE accumulated to 50% to 75% of B3URA3 (Figure 4B). However, B3URA3∆′3a accumulated to higher levels than both B3URA3 and B3URA3 + TRE in the absence of selection (Figure 4A) and in Ura^+^ colonies (Figure 4B). To determine the frequency of intramolecular RNA recombination in B3URA3 + TRE, 110 Ura^+^ colonies from three biological replicates were individually grown in 8-mL liquid cultures under uracil selection and total RNA was extracted and analyzed by northern blotting. Fifty-five percent of the Ura^+^ colonies maintained the initial B3URA3 + TRE replicon, 14 % harbored both B3URA3 + TRE and B3URA3∆′3a, and 31% lost B3URA3 + TRE and harbored only B3URA3∆′3a (Figure 4C). Accordingly, the frequency of intramolecular RNA recombination in B3URA3 + TRE was ~45% during the ~32 yeast generations needed to complete the experiment. Replacement of B3URA3 + TRE by B3URA3∆′3a could be explained by the higher replication of B3URA3∆′3a than B3URA3 + TRE (Figure 4A,B).

### 3.4. Recruitment into Replication Compartments Is Necessary for Intermolecular RNA Recombination 

As noted above, providing a transposed recruitment element within RNA3 was able to restore its 1a-mediated recruitment into the replication compartments (Figure 2). We hypothesized that a transposed recruitment element would also restore intermolecular RNA recombination in RNAs lacking a natural recruitment element. To test this hypothesis, a recombination experiment was conducted by co-expressing donor B3∆5′ with an acceptor RNA lacking the RNA3 intergenic region and harboring a transposed recruitment element (B3∆3′∆IR + TRE, Figure 3A). Acceptor RNAs lacking both the transposed and a natural recruitment element (B3∆3′∆IR), or harboring both a transposed and a natural recruitment element (B3∆3′ + TRE), and a set of constructs harboring a box B deletion in the natural or transposed recruitment element were also tested (Figure 3A,B). Parental RNAs were co-expressed in the presence of 1a and 2a^pol^ for nine yeast generations in the absence of uracil selection. After repressing transcription of parental RNAs, cells were plated on solid media lacking uracil. Uracil selection was used to identify cells harboring RNA3 replicons formed through recombination [10]. All Ura^+^ colonies detected were grown individually in 96-well plates, the RNA extracted, analyzed by northern blotting [43], and recombinants identified by size using a 5′3a probe (Figure 1B) [10]. The experiment was repeated three times with similar results.

**Figure 5:**
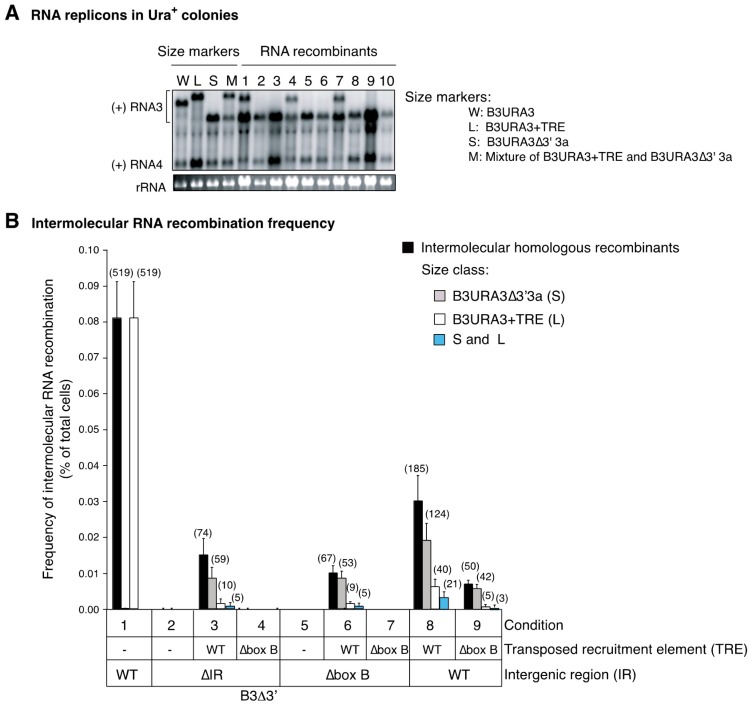
Intermolecular RNA recombinants from acceptor RNAs harboring a transposed recruitment element. (**A**) Sample Northern blot of intermolecular RNA recombinants between B3∆5′ and B3∆3′∆IR + TRE. B3URA3, B3URA3 + TRE (L), B3URA3∆3′3a (S), and a mixture of L plus S were used as size markers. Positive strand (+) RNA3 and RNA4 were detected with 32-P-labeled probes against URA3. Two size classes were identified. Short B3URA3∆3′3a (2, 3, 5, 6, 8, 9 and 10), long (L) B3URA3 + TRE in combination with B3URA3∆3′3a (1, 4 and 7) or alone. Ethidium bromide staining of 18S rRNA is indicated at the bottom. (**B**) Frequency of intermolecular RNA recombinants, organized by size, detected after transient induction of transcription of both B3∆5′ and B3∆3′ in nine conditions. In B3∆3′ the intergenic region was wild type (WT), deleted (∆IR), or had a box B deletion (∆box B). The transposed recruitment element (TRE) in B3∆3′ derivatives was absent (−), WT, or had a box B deletion. The histogram shows the average and standard error for three biological replicates. In parenthesis is the total number of cases observed in the same three biological replicates. On average the sample size per treatment was 210,000 cells.

RNAs lacking a functional recruitment element (Figure 3A,B) did not participate in intermolecular RNA recombination to detectable levels (Figure 5B, conditions 2 and 5). However, their ability to recombine was restored when a transposed recruitment element was provided (Figure 5B, conditions 3 and 6). In contrast, a box B-deficient transposed recruitment element failed to restore genomic RNA recruitment into replication compartments (Figure 2) and intermolecular RNA recombination (Figure 5B, conditions 4 and 7). The box B deletion removes 11 nt from the recruitment element (Figure 2A), is not expected to have an effect on the formation of crossover sites within 3a sequences and cause a 6% reduction in the random formation of crossover sites within the recruitment element. However, a box B deletion in the natural or in the transposed recruitment element resulted in the absence of recombinants (Figure 5B, conditions 4, 5, and 7). Accordingly, *cis*-acting sequences necessary for 1a-mediated recruitment to RNA replication compartments, even at a transposed location, are necessary and sufficient to promote intermolecular RNA recombination.

The role of the transposed recruitment element could be to provide 1a-mediated recruitment of genomic RNAs to replication compartments and/or to form crossover sites. To distinguish between these possibilities, we determined the frequency of structurally different B3URA3 + TRE and B3URA3∆3′3a replicons in Ura^+^ colonies (Figure 3C and Figure 5A).

Two distinct RNA products could be formed through recombination between donor B3∆5′ and acceptor B3∆3′∆IR + TRE based on the structure of the parental RNAs and the location of possible homologous crossover sites (Figure 3C). A longer product, B3URA3 + TRE, could be formed by crossing over within 3a sequences, while a shorter product, B3URA3∆3′3a, could be formed by crossing over from the intergenic recruitment element on B3∆5′ to the transposed recruitment element on B3∆3′∆IR + TRE (Figure 3C). B3URA3∆3′3a could also originate by intramolecular recombination between the natural and the transposed recruitment element after formation of B3URA3 + TRE (Figure 3C and Figure 4C). Crossovers exclusively between the natural and transposed recruitment elements of donor and acceptor would only form the shorter B3URA3∆3′3a replicon, and not the longer B3URA3 + TRE replicon (Figure 3C). Crossing over exclusively within 3′3a sequences common to donor and acceptor would form the longer B3URA3 + TRE product, which may be followed by formation of the shorter B3URA3∆3′3a product by intramolecular recombination (Figure 3C). 

To determine the nature of the recombinant replicons, RNA from all 74 Ura^+^ colonies recovered from three independent repetitions were analyzed by northern blotting. The recombinant replicons from ten randomly chosen samples are shown in Figure 5A. Three classes of Ura^+^ colonies harboring RNA recombinant progeny were detected: B3URA3∆3′3a was detected alone in 59 of the colonies (79.7%), B3URA3 + TRE alone in 10 (13.5%) or in combination with B3URA3∆3′3a in 5 (6.8%) (Figure 5B). Detection of the longer B3URA3 + TRE replicons in 15 of 74 (20.3%) Ura^+^ colonies (Figure 5B, condition 3) unambiguously shows the formation of recombinants by crossing over within 3′3a sequences common to the donor and acceptor RNAs without crossing over within the recruitment element (Figure 3C). The abundance of the B3URA3 + TRE replicon after prolonged selection is an under representation relative to initial B3URA3 + TRE formation because, as described in the previous section, B3URA3∆3′3a is derived by intramolecular recombination and can supplant B3URA3 + TRE (Figure 4C). These results rule out the exclusive formation of crossover sites within the recruitment element and show that the RNA recruitment element is not a preferred recombination site. These results are consistent with the recruitment element, and the box B, providing an essential function before the formation of crossover sites: the recruitment of individual parental RNAs into the replication compartments. Furthermore, these results indicate that intermolecular RNA recombination occurs in replication compartments receiving multiple RNAs. In this model, a limiting factor to intermolecular RNA recombination might be the availability of at least two genomic RNAs within reach of a template switch in the same replication compartment. 

For RNAs harboring a transposed recruitment element the frequency of recombination was 7- to 8-fold lower than that of acceptor B3∆3′, which harbors only the natural recruitment element (Figure 5B, compare condition 1 to 3 and 6). Similarly, when a complete (Figure 5B, condition 8) or a box B-deficient (Figure 5B, condition 9) transposed recruitment element was provided to B3∆3′, the frequency of intermolecular RNA recombinants was reduced 2.5-fold (Figure 5B, compare conditions 1 and 8) to 10-fold (Figure 5B, compare conditions 1 and 9). Replication under uracil selection was lower for RNA3 derivatives harboring a transposed recruitment element (Figure 4B), intermolecular RNA recombination is linked to replication, and identification of recombinants was based on replication-dependent uracil selection. Thus, the lower recombination efficiency of acceptors harboring a transposed recruitment element with respect to the acceptor harboring only a natural recruitment element may be related to the lower replication under selection of RNA3 derivatives harboring a transposed recruitment element (Figure 4B).

### 3.5. Regulating the Levels of Proteins 1a and 2a^pol^ Modulates the Type of Replication Compartments 

**Figure 6:**
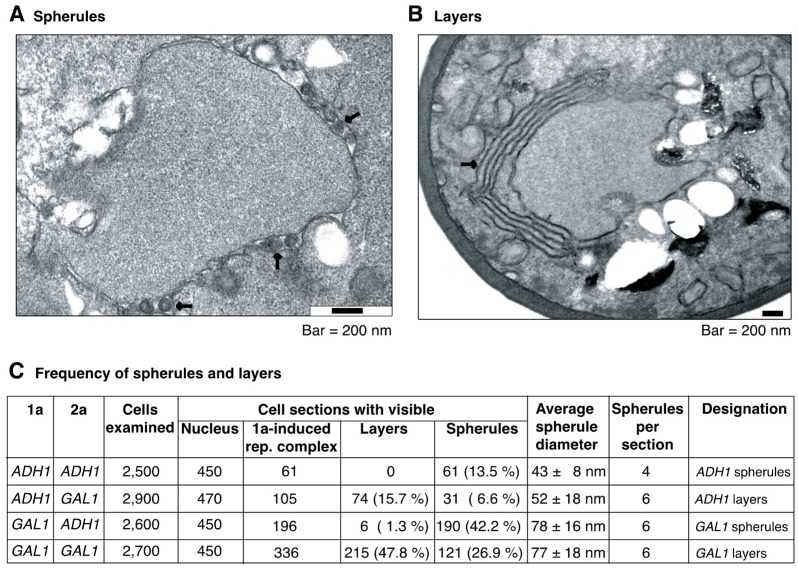
Alternate forms of BMV RNA replication compartments visualized by electron microscopy. (**A**) Arrows point to individual endoplasmic reticulum membrane-bound spherules; (**B**) The arrow points to rearrangement of the endoplasmic reticulum membrane in layers; (**C**) Expression of 1a and 2a^pol^ from *ADH1* or *GAL1* promoters and the resulting membrane rearrangements. Number of cells examined, number of cells with a visible nucleus, and the number of cells with visible BMV replication compartments, are indicated for each 1a and 2a^pol^ combination. The form of the replication compartment was classified as spherules or layers, and their relative abundance (%) is indicated with respect to the number of cells with visible nucleus. Within individual cells, spherules and layers were never detected together. The average spherule size and the average number of spherules per section is indicated. Spherules and layers were named based on the promoter driving expression of 1a.

Previous results in yeast showed that modulating the ratio of BMV replication proteins 1a and 2a^pol^ alters the type of replication compartment formed [27,36]. In the presence of 1a, a viral RNA template, and low levels of 2a^pol^, spherules are the predominant compartment made for BMV RNA replication [27]. However, by increasing the levels of 2a^pol^ and thus decreasing the 1a to 2a^pol^ ratio, there is a shift from spherular membrane rearrangements to large multilayer stacks of appressed double-membrane layers [36]. Double-membrane layers extend around half of the nucleus and thus occupy a larger surface area than individual spherules (Figure 6A,B), and the intermembrane space is not uniform but possesses some underlying variation [27,37]. Similar to spherules, layers are the site of 1a and 2a^pol^ accumulation, support RNA replication and protect RNA templates from nucleases [36].

Current estimates suggest that spherule volume is only large enough for one or two genomic RNA replication intermediates [20,27]. This suggests that a limiting factor to intermolecular RNA recombination is the lack of multiple viral RNAs within an individual spherular replication compartment, the absence of template switching, or both. This model predicts that a larger replication compartment would support RNA recombination to higher frequencies by sequestering multiple genomic RNAs and replication intermediates. To test this model, we first characterized the replication compartments induced by different levels of 1a and 2a^pol^ after expressing them from *ADH1* or *GAL1* promoters, in all possible combinations (Figure 6C). 

**Figure 7:**
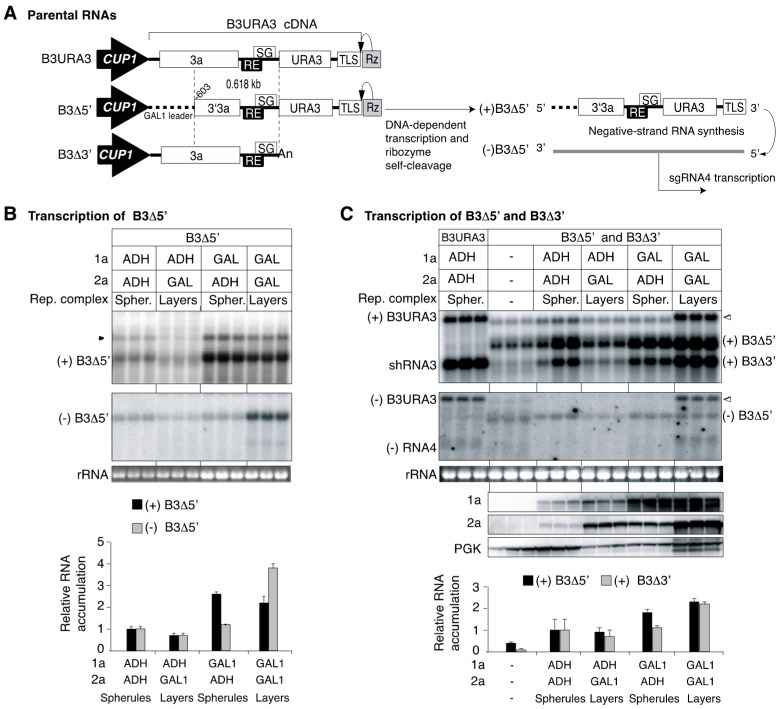
Accumulation of parental B3Δ5′ and B3Δ3′ in spherules and layers. (**A**) Features of *CUP1*-driven B3Δ5′ and B3Δ3′ in relation to B3URA3. Labels are as in Figure 1A. Illustration of DNA-dependent transcription of B3Δ5′, (−) negative-strand RNA synthesis and subgenomic RNA4 transcription. (**B**) Accumulation of positive (+)- and negative (−)-strand B3Δ5′ after transient transcription for nine yeast generations (72 to 96 h) in liquid cultures containing galactose and 500 μM CuSO4. 1a and 2a^pol^ were expressed from the *ADH1* or the *GAL1* promoter. The form of the BMV replication complex induced is indicated. Equal amounts of cells were harvested, and equal amounts of RNA analyzed by Northern blotting. Positive (+)- and negative (−)- strand RNA was detected using a using a 32-P labeled probe targeting 3′3a or URA3, respectively. The black arrowhead points to transcripts that have not been cleaved by the ribozyme. Ethidium bromide staining of 18S rRNA is indicated at the bottom. The histogram illustrates B3Δ5′ accumulation in each condition relative to *ADH1* spherules. Bars represent the average and standard error of three biological replicates. Positive-strand B3Δ5′ accumulated to higher levels (*p* < 0.01) in *GAL1* spherules and *GAL1* layers than in other conditions. Negative-strand B3Δ5′ accumulated to higher levels (*p* < 0.01) in *GAL1* layers that in other conditions. (**C**) Accumulation of positive (+)- and negative (−)-strand B3Δ5′ and B3Δ3′, and replication proteins. Cultures were grown and samples were processed as in (**B**) except that duplicate samples were collected for total RNA or protein extraction. B3URA3 was included as size marker. (+) B3URA3 and (-) B3URA3 indicate B3URA3 of positive and negative polarity, respectively. shB3URA3 is a transcript formed after premature termination of transcription at the oligo (A) tract (Sullivan and Ahlquist, 1999) and is approximately the same size as B3Δ3′. Empty arrowheads point to intermolecular RNA recombinants matching the size of B3URA3. The middle panel shows accumulation of replication proteins 1a and 2a^pol^ in conditions inducing the formation of spherules or layers. 1a and 2a^pol^ were detected by immunoblotting, and 3-phosphoglycerate kinase (PGK) was used as loading control. The histogram illustrates B3Δ5′ or B3Δ3′ accumulation in each condition relative to *ADH1* spherules. Bars represent the average and standard error of three biological replicates. Positive-strand B3Δ5′ accumulated to higher levels (*p* < 0.01) in GAL1 spherules and GAL1 layers that in other conditions. Positive-strand B3Δ3′ accumulated to higher levels (*p* < 0.01) in GAL1 layers than in other conditions.

Two conditions predominantly formed spherules (Figure 6A,C): *ADH1*-driven 1a with *ADH1*-driven 2a^pol^ (*ADH1* spherules described above) and *GAL1*-driven 1a with *ADH1*-driven 2a^pol^ (*GAL1* spherules). However, spherules formed when 1a was expressed from the *GAL1* promoter were nearly twice the size (~78 nm) and found in ~3-fold more cells than spherules formed when 1a was expressed from the weaker *ADH1* promoter (Figure 6C). *GAL1* is a stronger promoter than *ADH1* and thus more 1a protein is available to form larger and more abundant spherules (Figure 7C), and variation in spherule size has been previously reported under various experimental conditions [31].

Likewise, two conditions resulted predominantly in layer formation (Figure 6B,C): *ADH1*-driven 1a with *GAL1*-driven 2a^pol^ (*ADH1* layers), and *GAL1*-driven 1a with *GAL1*-driven 2a^pol^ (G*AL1* layers). These results are in agreement with previous studies that characterized layer formation upon expression of both 1a and 2a^pol^ from the *GAL1* promoter [27]. Although the number of double-membrane layers did not vary significantly, layers were present in ~3-fold more cells when 1a was expressed from the *GAL1* promoter (Figure 6C). These results are consistent with higher accumulation of 1a (Figure 7C), the 1a-dependent formation of replication structures, and the 2a^pol^-dependent modulation of the shape of the replication structures [27,37]. In three of the four conditions tested a mixture of cell sections containing spherules or layers was detected (Figure 6C). However, within the same cell, spherules and layers were never detected together. 

The results described above show that BMV replication in yeast allows the experimental manipulation of the type, size and abundance of RNA replication compartments and is an ideal experimental system to gain insight on the role of the structure of the RNA replication compartment in intermolecular RNA recombination.

### 3.6. Intermolecular RNA Recombination Occurs at Higher Frequency in Layers Than in Spherules

As double-membrane layers provide a larger surface than individual spherules (Figure 6) [27,37], we hypothesized that layers support intermolecular RNA recombination at higher frequency than spherules by providing a larger space to accommodate multiple genomic RNAs. To test this model, a recombination experiment was conducted in which parental RNAs B3∆5′ and B3∆3′ were co-expressed under conditions inducing the formation of spherules or layers. Yeast were transformed with plasmids encoding one (B3∆5′) or both parental RNAs (B3∆5′ and B3∆3′, Figure 7A) from the *CUP1* promoter. Replication proteins 1a and 2a^pol^ were expressed from the *ADH1* or *GAL1* promoter in various combinations to induce the formation of spherules or layers (Figure 6C). Accumulation of parental RNAs B3∆5′ and B3∆3′ was determined by northern blotting after transient induction of transcription for nine yeast generations (Figure 7B). Strikingly, when 1a and 2a^pol^ were expressed from the *GAL1* promoter (layer-forming conditions), intermolecular recombinant RNA was detected in addition to parental RNA transcripts even before plating and selection (Figure 7C). Such RNA was not detected when B3∆5′ was transcribed in the absence of B3∆3′ (Figure 7B), indicating that *GAL1*-layers support intermolecular RNA recombination at high frequency. 

**Figure 8:**
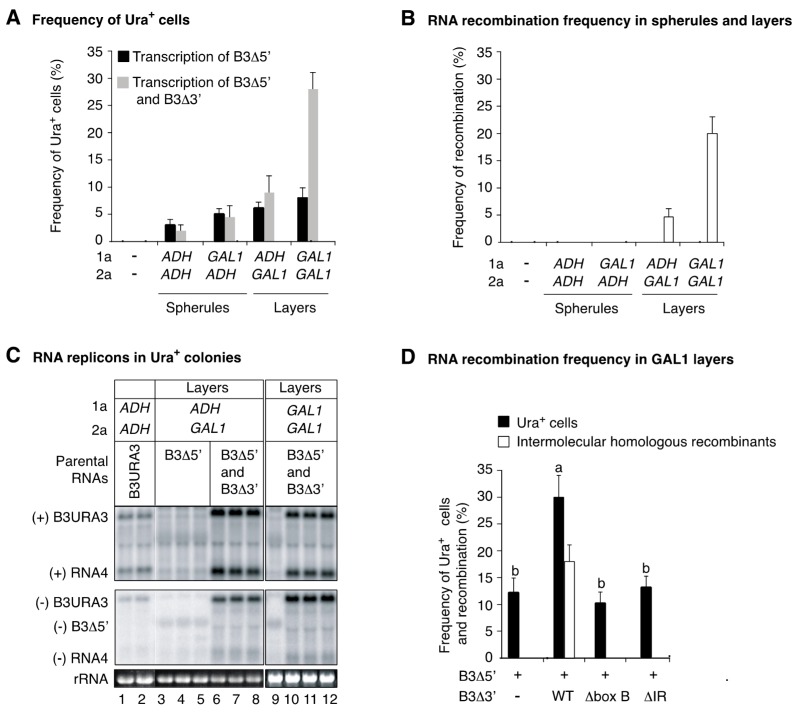
Intermolecular RNA recombination in layers after one yeast generation. Cells were incubated for 15 h in liquid media (8 mL) containing galactose and 500 μM CuSO4. 1a and 2a^pol^ were expressed from the *ADH* or *GAL1* promoters to induce the formation of spherules or layers. Ura^+^ cells were identified by uracil selection on plates. The average sample size was 700 cells per treatment. (**A**) Frequency of Ura^+^ cells after transcription of B3Δ5′ alone or in combination with B3Δ3′. Transcription of B3Δ5′ alone supported Ura^+^ colony formation by directing RNA4 transcription. A significant (*p* < 0.01) increment in the number of Ura^+^ cells was obtained in layer-forming conditions. (**B**) Frequency of intermolecular RNA recombination in Ura^+^ cells described in (**A**). (**C**) The presence of intermolecular RNA recombinants was confirmed by analyzing 48 Ura^+^ colonies individually grown for 36 h in cultures (8 mL) lacking uracil and copper. RNA was extracted an analyzed by Northern blotting using URA3 probes. Representative blots are shown. B3URA3 was a size marker (lanes 1 and 2). (+) and (−) indicate RNA of positive or negative polarity, respectively. As illustrated in Figure 7A, B3Δ5′ supported negative-strand B3Δ5′ synthesis and RNA4 transcription (lanes 3, 4, 5 and 9). Lanes 6, 7, 8, 10, 11 and 12 are intermolecular RNA recombinants. (**D**) Frequency of Ura^+^ cells obtained after transient transcription of B3Δ5′ and wild type or mutant (box B or intergenic region deletion) B3Δ3′ for one yeast generation in *GAL1* layers. Induction of transcription, selection, and identification on intermolecular RNA recombinants was as in (**A**,**B**). Bars represent the average and standard error of three biological replicates. Bars with the same letter are not significantly different (Tukey's test with alpha = 0.01).

To determine the nature of the RNA supporting Ura^+^ colony formation (Figure 8A), total RNA from yeast expressing various parental RNA combinations that induced the formation of spherules or layers was extracted from 40 individual colonies and analyzed by northern blotting using a URA3 probe. We failed to detect small sURA3 replicons formed through intramolecular RNA recombination [10], and only detected negative-strand B3∆5′ (Figure 8C). Thus, Ura^+^ colonies detected upon transcription of donor B3∆5′ from the *CUP1* promoter were mainly the result of leaky transcriptional activity amplified by synthesis of negative-stand B3∆5′ and subgenomic RNA4 transcription (Figure 7A,B).

The sections used for EM analysis were 70 nm thick; there were between 4 and 6 spherules per cell section (Figure 6C). Accordingly, there are between 170 and 225 spherules per cell. However, the frequency of BMV RNA recombination per cell was 0.08% (Figure 1B). For both spherule forming conditions, similar levels of Ura^+^ cells were detected after transient transcription of B3∆5′ alone or in combination with B3∆3′ (less than 5%, Figure 8A), and no intermolecular RNA recombinants were identified by northern blotting (Figure 8B). These results are consistent with previous findings showing that in *ADH1* spherules, intermolecular RNA recombinants are formed at a frequency of 1.7 × 10^−4^ per cell per yeast generation [10]. In experiments described here, the average sample size was 700 cells, which is far below the detection limit of the plating assay used.

A consistent increment in the frequency of Ura^+^ cells was detected when both parental RNAs (B3∆5′ and B3∆3′) were transcribed in layer forming conditions (Figure 8A). In Ura^+^ colonies the presence of intermolecular RNA recombinants was confirmed by northern blotting using a URA3 probe (Figure 8C). The frequency of intermolecular RNA recombination was calculated as the difference between the number of Ura^+^ cells detected after transcription of both B3∆5′ and B3∆3′ minus the number of Ura^+^ cells detected after transcription of B3∆5′ alone, as before [10]. On a per-cell basis, on average, the frequency of intermolecular RNA recombination was 20% in *GAL1* layers and 5% in *ADH1* layers (Figure 8B). This difference correlated with a higher number of cell sections harboring *GAL1* layers (47.8%) than *ADH1* layers (15.7%) (Figure 6C), and with higher accumulation of 1a (Figure 7C). Protein 1a accumulated to similar levels in *GAL1* layers and *ADH1* spherules (Figure 7C). Thus, the difference in intermolecular RNA recombination frequency is independent from 1a. 

After one yeast generation, comparing the recombination frequency observed in layers (Figure 8B) to that in spherules (1.7 × 10^−4^ per cell) (Figure 1) [10] shows that intermolecular RNA recombination occurred at a dramatically higher frequency in layers (~230-fold for *ADH1* layers and ~1170-fold for *GAL1* layers) than in spherules. Electron microscopy analysis showed that the number of cell sections harboring *GAL1* spherules (42.2%) was higher than the number of cells harboring *ADH1* layers (15.7%) (Figure 6C). Contrary to the frequency of intermolecular RNA recombination, both parental RNAs B3∆5′ and B3∆3′ accumulated to higher levels in *GAL1* spherules than in *ADH1* layers (Figure 7C). Furthermore, 1a accumulated to similar levels in *GAL1* spherules and *GAL1* layers (Figure 7C). 2a^pol^ accumulated 1.5 to two-fold higher in layers than in spherules (Figure 7C). 

Results described above show that layers are more permissive to intermolecular RNA recombination than spherules. The low frequency of intermolecular RNA recombinants in spherules cannot be explained by a low number of cells harboring spherular replication compartments, by low abundance of parental RNAs, or by reduced levels of viral RNA polymerase. 

### 3.7. The Template Recruitment Element Is Required for Intermolecular RNA Recombination in Layers 

RNA recombination occurred at higher frequency in layers than in spherules (Figure 8B), and recruitment of genomic RNAs into layers requires the recruitment element [37]. We hypothesized that formation of intermolecular RNA recombinants in layers requires a functional recruitment element on both parental RNAs. To test this hypothesis, donor RNA B3∆5′ was expressed alone or in combination with a wild type or recruitment element-deficient B3∆3′ (box B or intergenic region deletion). To induce layer formation, both 1a and 2a^pol^ were expressed from the *GAL1* promoter (Figure 6C). Transcription of parental RNAs was induced from the *CUP1* promoter for one yeast generation (Figure 6C). As described above (Figure 8A), transcription of B3∆5′ alone supported Ura^+^ colony formation mediated by transcription of subgenomic RNA4 (Figure 7A). Presence of intermolecular RNA recombinants in the progeny was confirmed by northern blotting (Figure 8C).

A three-fold increment in the frequency of Ura^+^ colonies was observed when wild type B3∆3′ was co-expressed with B3∆5′ (Figure 8D), and intermolecular RNA recombinants were detected only when wild type B3∆3′ was co-expressed with B3∆5′. No intermolecular RNA recombinants were detected when the recruitment element was removed from B3∆3′ or harbored a box B deletion (Figure 8D). Thus, a box B or an intergenic region deletion on B3∆3′ reduced the frequency of intermolecular RNA recombination below the detection limit (Figure 8D). These results show that formation of intermolecular RNA recombinants in layers, as in spherules, requires a functional recruitment element on both parental RNAs.

## 4. Discussion

During intermolecular RNA recombination by strand switching, parental RNAs must be within reach of the RNA polymerase and nascent strand complex as in HIV virions, which contain an RNA dimer formed in the cytoplasm before the RNA is packaged into the newly formed viral particle [45]. However, HIV-1 Gag recognizes one dimeric RNA and not two monomeric RNA molecules [46]. Recruitment of two or more BMV genomic RNAs into replication compartments might occur through several alternate mechanisms, such as dimerization or multimerization by association of genomic RNAs with each other prior to 1a-mediated recruitment into the replication compartments; non-specific, *cis*-acting element-independent recruitment [47]; near simultaneous interaction of two genomic RNAs with a single 1a protein fortuitously resulting in co-recruitment; or individual recruitment of multiple viral genomic RNAs into a single replication compartment. Additionally, intermolecular RNA recombinants could be formed outside replication compartments. Our results provide two lines of evidence against the formation of genomic RNA multimers prior to 1a-mediated recruitment, non-specific recruitment in a 1a-independent manner, or fortuitous co-recruitment. First, without being part of crossover sites (Figure 1 and Figure 5), formation of intermolecular RNA recombinants required a functional recruitment element on both parental RNAs in both spherules (Figure 5) and layers (Figure 8D). Second, if intermolecular RNA recombination or dimerization of genomic RNAs occurred outside replication compartments, the frequency of intermolecular RNA recombination would be independent of the form of the replication compartment. However, by changing the form of the replication compartments from spherules to layers, the frequency of RNA recombination increased 200- to 1000-fold (compare Figure 1C and Figure 8B). Thus, parental RNAs do not form dimers and intermolecular RNA recombinants are not formed outside replication compartments. 

BMV RNA replication occurs on ER membranes inside vesicle-shaped, spherular RNA replication compartments [20,27] that are very similar to the replication compartments of other plant and animal viruses [20,21,22,35,36]. BMV RNA recombination occurs predominantly as a byproduct of RNA replication, mainly by polymerase-driven template switching [6,7,10], occurs at similar frequencies in plants and in yeast [10,48], and both parental RNAs require a functional template recruitment element (Figure 5) [10]. Distribution of crossover sites in intermolecular RNA recombinants using point mutations and structurally different parental RNAs showed that the template recruitment element is not a preferred recombination site (Figure 1 and Figure 5). The recruitment element provides *cis*-acting signals indispensable for the 1a-dependent selection and recruitment of parental RNA into replication compartments (Figure 2) [26,27]. Accordingly, a box B or a recruitment element deletion abolished both template recruitment and intermolecular RNA recombination both in spherules and in layers (Figure 5 and Figure 8D). Interestingly, both template recruitment and intermolecular RNA recombination were restored by providing a transposed recruitment element and a fraction of the recombinants formed by crossing over outside the recruitment element (Figure 5). These results support a model in which formation of intermolecular RNA recombinants occurs mainly after parental RNAs are individually recruited into the replication compartments through their respective recruitment elements (Figure 5 and Figure 8D) and rules out the formation of BMV RNA recombinants outside the RNA replication compartments.

The frequency of intermolecular RNA recombination in plant cells [48] is similar to that observed in yeast under spherule-forming conditions (Figure 1) [10]. Spherules are the natural form of the replication compartments of many plant positive-strand RNA viruses, including BMV [20,21,35,36], and individual spherules are large enough for one or two genomic RNA replication intermediates [20,27]. Thus, compartmentalization of RNA replication in spherules could be a limiting factor to intermolecular RNA recombination. This model predicts that expanding the size of the replication compartments would remove the physical constrain that limits the number of viral RNAs that can be recruited, which in turn would lead to a higher frequency of intermolecular RNA recombination. In support of this model, intermolecular RNA recombination frequency in layers was 200- (*ADH* layers) to 1000-fold (*GAL1* layers) (Figure 8B) higher than that observed in conditions forming spherules (Figure 1C). This difference is not related to any aspects of RNA replication, including the number of spherules or layers per cell (Figure 6) or the abundance of parental RNA transcripts (Figure 7B,C). Higher levels of 2a^pol^ associated with layer formation might promote template switching (Figure 7C), however, the ~2-fold increase in 2a^pol^ accumulation associated with layer formation (Figure 7C) [37] appears unlikely to explain the dramatic increase in recombination frequency. In support of this conclusion, 2a^pol^ levels were only 1.2-fold higher in *ADH* layers that in GAL1 spherules (Figure 7C), but RNA recombination frequency in *ADH* layers was >200 fold higher (Figure 8). 

The difference in intermolecular RNA recombination frequency between spherules and layers suggest that their ultra-structure is different in one or more key elements that regulate intermolecular RNA recombination. A model to explain the difference is that layer organization favors two fundamental parts of the recombination process [49]: aggregating multiple genomic RNAs within close proximity, provide an environment that enhances template switching, or both. Both conditions occur in retrovirus virions, which are diploid [45], and support intermolecular RNA recombination at frequencies of 10% to 40% [50,51], which is similar to BMV intermolecular RNA recombination in layers (Figure 8B). The BMV RNA polymerase is capable of crossing over in spherules, as indicated by the high frequency of intramolecular RNA recombination (Figure 4C). What is the basis for the strong restriction of RNA recombination in spherules but not in layers? One way to explain such restriction is that spherules receive only one viral genomic RNA, and only occasionally two or more. Perhaps even the recombination events detected in spherule-forming conditions might be due to some aberrant form of the replication compartments. 

Enhanced RNA recombination was detected for Tomato bushy stunt virus (TBSV) in a yeast strain lacking Pmr1p, an ion pump that controls Ca^2+^/Mn^2+^ into the Golgi from the cytosol [52]. Yeast lacking Pmr1p supported higher TBSV replication and recombination than wild type cells, and both effects were attributed to higher accumulation of Mn^2+^ in the replication compartments [52]. Similarly, reduced NTP concentration induces template switching [49]. We cannot rule out the possibility that layers could maintain a higher ion balance or lower NTP concentration than spherules. However, that is likely not the case, because both layers and spherules support BMV replication to similar levels [37].

Poliovirus, a positive-strand RNA virus, undergoes homologous recombination at a frequency estimated to be as high as 10^−1^–10^−2^ recombination events per genome [53]. Early in infection a high percentage (>85%) of individual poliovirus replication complexes contained at least two parental RNAs [54]. Electron tomography reconstructions show that the highest rate of viral RNA synthesis, which is when the majority of RNA recombination occurs, takes place in single membrane convoluted tubules that are closer in structure to the BMV-induced layers than spherules [39]. Thus, for plant and animal positive-strand RNA viruses, the form of the viral RNA replication compartment might determine the frequency of intermolecular RNA recombination.
